# Inflammation scores based on C-reactive protein and albumin predict mortality in hospitalized older patients independent of the admission diagnosis

**DOI:** 10.1186/s12979-024-00471-y

**Published:** 2024-10-09

**Authors:** Mirko Di Rosa, Jacopo Sabbatinelli, Angelica Giuliani, Miriam Carella, Daniele Magro, Leonardo Biscetti, Luca Soraci, Francesco Spannella, Massimiliano Fedecostante, Federica Lenci, Elena Tortato, Lorenzo Pimpini, Maurizio Burattini, Sara Cecchini, Antonio Cherubini, Anna Rita Bonfigli, Maria Capalbo, Antonio Domenico Procopio, Carmela Rita Balistreri, Fabiola Olivieri

**Affiliations:** 1 Centre for Biostatistics and Applied Geriatric Clinical Epidemiology, IRCCS INRCA, Ancona, Italy; 2 Department of Clinical and Molecular Sciences, Università Politecnica Delle Marche, Ancona, Italy; 3 Clinic of Laboratory and Precision Medicine, IRCCS INRCA, Ancona, Italy; 4 Istituti Clinici Scientifici Maugeri IRCCS, Cardiac Rehabilitation Unit of Bari Institute, Bari, Italy; 5 Complex Operative Unit of Clinical Pathology, ARNAS Civico Di Cristina e Benfratelli Hospitals, Palermo, Italy; 6 Cellular, Molecular and Clinical Pathological Laboratory, Department of Biomedicine, Neuroscience and Advanced Diagnostics (Bi.N.D.), University of Palermo, Palermo, Italy; 7 Unit of Neurology, IRCCS INRCA, Ancona, Italy; 8 Unit of Geriatric Medicine, IRCCS INRCA, Cosenza, Italy; 9 Internal Medicine and Geriatrics, IRCCS INRCA, Ancona, Italy; 10 Geriatria, Accettazione Geriatrica e Centro Di Ricerca Per L’invecchiamento, IRCCS INRCA, Ancona, Italy; 11 Unit of Nephrology and Dialysis, IRCCS INRCA, Ancona, Italy; 12 Diabetology Unit, IRCCS INRCA, Ancona, Italy; 13 Cardiology Unit, IRCCS INRCA, Ancona, Italy; 14 Internal Medicine Department, IRCCS INRCA, Osimo, Italy; 15 Diagnostic Imaging, Clinical and Interventional Radiology, IRCCS INRCA, Osimo, Italy; 16 Scientific Direction, IRCCS INRCA, Ancona, Italy; 17 IRCCS INRCA, Ancona, Italy; 18 Advanced Technology Center for Aging Research, IRCCS INRCA, Ancona, Italy

**Keywords:** Older patients, In-hospital mortality, C-reactive protein, CRP-to-albumin ratio

## Abstract

Systemic inflammation significantly increases the risk of short- and long-term mortality in geriatric hospitalized patients. To predict mortality in older patients with various age-related diseases and infections, including COVID-19, inflammatory biomarkers such as the C-reactive protein (CRP) to albumin ratio (CAR), and related scores and indexes, i.e. Glasgow Prognostic Score (GPS), modified GPS (mGPS), and high sensitivity (hs)-mGPS, have been increasingly utilized. Despite their easy affordability and widespread availability, these biomarkers are predominantly assessed for clinical purposes rather than predictive applications, leading to their underutilization in hospitalized older patients.

In this study, we investigated the association of CAR, GPS, mGPS, and hs-mGPS with short-term mortality in 3,206 geriatric hospitalized patients admitted for acute conditions, irrespective of admission diagnosis. We observed that unit increases of CAR, and the highest classes of GPS, mGPS, and hs-mGPS were significantly associated with a two- to threefold increased risk of death, even adjusting the risk for different confounding variables. Interestingly, a hs-mGPS of 2 showed the highest effect size. Furthermore, gender analysis indicated a stronger association between all CRP-albumin based parameters and mortality in men, underscoring the gender-specific relevance of inflammation-based circulating parameters in mortality prediction.

In conclusion, scores based on serum CRP and albumin levels offer additional guidance for the stratification of in-hospital mortality risk in older patients by providing additional information on the degree of systemic inflammation.

**Supplementary Information:**

The online version contains supplementary material available at 10.1186/s12979-024-00471-y.

## Introduction

The aging phenomenon represents one of the most significant challenges of our time. The demographic group of the oldest old individuals (aged 85 years and above) is notably the fastest-growing, particularly in developed nations (https://www.who.int/news-room/fact-sheets/detail/ageing-and-health). This will lead to a subsequent increase in the number of people affected by age-related diseases (ARDs) and geriatric syndromes, i.e. frailty, sarcopenia, functional disability, cognitive impairment, multimorbidity, and poor nutritional status, along with a significant rise in care burden and expenses due to hospitalizations [1–3]. In addition, hospitalized older patients also show a high rate of short- and long-term mortality [4]. Consequently, it is crucial to predict these syndromes, assess their severity, and anticipate the factors that may influence in-hospital mortality, to inform clinical decision-making and treatment strategies. Many scores and indexes have been used until now to achieve this aim, and among these, it is possible to include the Clinical Frailty Scale (CFS) [5], Charlson Comorbidity Index (CCI) [6], and Multidimensional Prognostic Index (MPI) [7]. Systemic inflammation is well recognized as a prognostic factor for its role in modulating both the severity of geriatric syndromes and associated complications, such as prolonged hospitalization and care, and ultimately death [8, 9]. In particular, scores encompassing the inflammation marker C-reactive protein (CRP) and albumin, which reflect innate immunity activation, liver function, and nutritional status [10], are widely used due to their affordability and widespread availability [11].

The CRP-to-albumin ratio (CAR) and its related categorical scores, including the Glasgow Prognostic score (GPS), modified Glasgow prognostic score (mGPS), and high-sensitivity-mGPS (hs-mGPS), all linked to the relationship between the plasma levels of CRP and albumin, respectively, have been assessed to predict mortality in older patients affected by many ARD and infections, i.e. Coronavirus disease 19 (COVID-19) [12–16]. While mGPS attributes a 10 mg/L serum CRP cutoff, which mostly reflects overt systemic inflammation, hs-mGPS is based on a more stringent threshold, i.e. 3 mg/L, which has been extensively associated with heightened cardiovascular risk due to residual also in asymptomatic individuals [17].

Since CAR and its derivatives are employed as tools for monitoring cancer-related cachexia, most of the evidence supporting their prognostic value has been gathered in patients affected by various types of cancer [18]. In this framework, scores based on albumin and CRP are often evaluated in conjunction with other biomarkers that reflect systemic inflammation, such as indexes derived from the complete blood cell count, including neutrophil-to-lymphocyte ratio (NLR) [19]. Data supporting a broader diagnosis-independent use in hospitalized patients is limited. The use of CRP/albumin-based scores holds potential in the setting of older patients, as hypoalbuminemia, which is remarkably prevalent in this population, has already been identified as an unfavorable prognostic marker [20]. Moreover, these scores may provide additional guidance in the characterization of organ dysfunction in older patients [21]. To the best of our knowledge, only one study has investigated the role of GPS on short- and long-term mortality in 529 hospitalized older patients affected by multimorbidity, demonstrating that a GPS score of 2 represents an independent factor significantly associated with 6-month and 1-year mortality in these patients [22]. However, limited data are available on the prognostic value of these scores for predicting adverse hospitalization outcomes.

Here, we examined the association of CAR, GPS, mGPS, and hs-mGPS with short-term mortality in a large cohort of older patients hospitalized due to acute conditions and assessed their prognostic relevance independent of admission diagnosis. Additionally, because previous data indicated that systemic inflammation might have a greater impact on all-cause mortality in males compared to females [23], we performed a sensitivity analysis in this study to account for sex-related differences potentially affecting the association of CAR, GPS, mGPS, and hs-mGPS with the outcome.

## Methods

### Study design and population

In this study, we used data derived from the Report-AGE project, a large-scale observational study on the health conditions of older adult patients (over 65 years) admitted to the acute care wards of the Italian National Institute of Health and Science on Ageing (IRCCS INRCA) [24].

Briefly, all patients consecutively admitted to participating wards from September 2011 to October 2021 were asked to participate. In the case of a participant’s cognitive decline or poor capability of judgment, a proxy (relative or caregiver) was invited to give consent in addition to obtaining consent from the participant. After obtaining written informed consent from the patient or caregiver, all patients underwent comprehensive geriatric assessment (CGA) by Inter-RAI Minimum Data Set acute care (MDS-AC) [25] performed both at the time of hospitalization and at discharge. Criteria for inclusion were age ≥ 65 years, length of stay more than 24 h, and signed informed consent. Only patients affected by Coronavirus disease-19 (COVID-19) were excluded, due to the very high mortality rate (about 40% during the first waves of COVID-19 pandemic in 2020–2021) associated with the disease in the context of a geriatric hospital, which could represent a potentially relevant confounding factor. Routine laboratory parameters were available for 3,206 hospitalizations (2,426 subjects and 780 repeated hospitalizations). Participating physicians and nurses received specific before recruitment, as previously described [26]. Data on disease and medications history of all patients recruited for this study were obtained from the medical records. The main acute and chronic diseases at hospital admission were coded in accordance with the International Classification of Diseases, 9th revision (http://www.icd9data.com/). Medications were recorded according to their Anatomical Therapeutic Chemical (ATC) codes. The degree of disability was assessed using the Katz-15 Index of Independence in Basic Activities of Daily Living (ADL) and Instrumental Activities of Daily Living (IADL) [27].

The Ethical Committee of the Italian National Research Center on Aging has approved the study protocol which was in accordance with the 1964 Helsinki Declaration. The trial registration number is NCT0139768 2. Signed informed consent was obtained from all participants in the study.

### Outcome

Outcome measure of interest was in-hospital death. Patients were followed from hospital admission until died in hospital or discharged alive. Length of hospital stay was calculated as the time from the patient’s admission to the acute care unit until discharged or died in the hospital.

### Study variables

Complete blood count and serum concentrations of alanine aminotransferase (ALT), ferritin, and creatinine were assessed by standard procedures. The neutrophil-to-lymphocyte ratio was calculated as the ratio of neutrophil and lymphocyte absolute counts. The glomerular filtration rate (GFR) was estimated by the creatinine-based Berlin Initiative Study -1 (BIS1) equation specially used to estimate GFR in older adults [28].

Serum albumin and CRP were measured using the Cobas® ALB2 colorimetric assay and the Cobas® CRPL3 immunoturbidimetric assay, respectively, on a Roche Cobas® c501 clinical chemistry analyzer. The detection range for the CRP assay was 0.3 – 350.0 mg/L.

The traditional GPS is derived by allocating one point each for elevated CRP (> 10 mg/L) and hypoalbuminemia (serum albumin < 35 g/L), so that patients with both, one, or neither of these conditions would have a score of 2, 1, or 0, respectively [29]. For the modified GPS (mGPS), patients with hypoalbuminemia were assigned a score of 0 in the absence of an elevated C-reactive protein. For the high-sensitivity modified GPS (hs-mGPS), 3 mg/L (rather than 10 mg/L) is used as the CRP cut-off value. The detailed criteria of systemic inflammation-based prognostic scores, mGPS, and hs-mGPS are reported in Table 1.

**Table 1:** Criteria for determining GPS, mGPS, and hs-mGPS

Prognostic score	Criteria	Score allocated
**GPS**	CRP ≤ 10 mg/L and Alb ≥ 35 g/L	0
	CRP > 10 mg/L or Alb < 35 g/L	1
	CRP > 10 mg/L and Alb < 35 g/L	2
**mGPS**	CRP ≤ 10 mg/L	0
	CRP > 10 mg/L and Alb ≥ 35 g/L	1
	CRP > 10 mg/L and Alb < 35 g/L	2
**hs-mGPS**	CRP ≤ 3 mg/L	0
	CRP > 3 mg/L and Alb ≥ 35 g/L	1
	CRP > 3 mg/L and Alb < 35 g/L	2

*mGPS* Modified Glasgow prognostic score, *hs-mGPS* High-sensitivity modified Glasgow prognostic score, *CRP* C-reactive protein, *Alb* Albumin

### Data analysis

Medians with interquartile range were used to describe continuous variables with non-normal distribution (assessed with the Shapiro–Wilk test); absolute frequencies and percentages were used for categorical variables. The Chi-square test and the Wilcoxon rank-sum test were performed to compare variables between groups (survived vs deceased patients), as appropriate. GPS, mGPS, and hs-mGPS associations with in-hospital mortality were evaluated by using Kaplan-Meier curves and statistical significance was assessed using Log-rank tests for equality of survival functions. In order to identify the CAR cutoff of increased risk in our population, we applied the Receiver operating characteristics (ROC) curve analysis with Youden’s method to maximize the sum of sensitivity and specificity. The association of CAR, GPS, mGPS and hs-mGPS with in-hospital mortality was then assessed by using univariate and multivariate Cox regression models for each index: Model 1 was unadjusted; Model 2 was adjusted for age and gender; Model 3 was adjusted for age, gender, use of 5 or more drugs, loss of 3 or more ADL or IADL, eGFR (BIS1), NLR, ALT, Hgb, platelets, acute myocardial infaction (AMI), congestive heart failure (CHF), cerebrovascular disease (CeVD), dementia, chronic obstructive pulmonary disease (COPD), Parkinson’s disease, hypertension, chronic kidney disease (CKD), liver cirrhosis, diabetes and cancer. Tests of proportional hazards assumption using Schoenfeld residuals were performed for each Cox regression model and all of them accepted the null hypothesis (*p* > 0.05). Akaike and Bayesian information criteria (AIC and BIC) were computed to compare the model fit of Cox regressions. The continuous, category-free, net reclassification improvement (NRI) was used to assess the increment of predictive value for the combination of NLR and CAR [30]. A bootstrap procedure with 1000 replications was used to estimate a 95% Confidence Interval (CI) for the area under the curve (AUC) and NRI. To account for sex differences, Kaplan-Meyer curves and Cox regression Models were repeated separating males from females. All the analyses were performed with Stata/MP 18.0 (StataCorp LP, CollegeStation, TX, USA). P values less than 0.05 (two-sided) were considered statistically significant.

## Results

Demographic, clinical and laboratory characteristics collected at the time of hospital admission of subjects included in the present analysis are reported in Table 2. Overall, the 3,206 patients included in the study had a median age of 88 (84–92) years, were predominantly women (58.1%), and the median length of hospital stay was of 9 (IQR 6 – 13, range 1 – 25) days. During the hospital stay, 450 out of 3,206 (14%) hospitalizations ended with death. As expected, patients who died were significantly older, had a higher prevalence of sepsis and several chronic diseases, including CHF, COPD, hypertension, CKD, diabetes, and cancer compared to those who survived. All the CRP/albumin-based scores, i.e. CAR, GPS, mGPS, and hs-mGPS, were significantly higher (*p* < 0.001 for all) in deceased patients.

**Table 2:** Sample characteristics

	**Total**	**Survived**	**Deceased**	***p*-value**
	***N* = 3,206**	***N* = 2,756**	***N* = 450**	
**Age (years)**	88 (84–92)	87 (84–91)	89 (86–93)	**< 0.001**
**Gender, n (%)**				0.878
** M**	1,343 (41.9%)	1,153 (41.8%)	190 (42.2%)	
** F**	1,863 (58.1%)	1,603 (58.2%)	260 (57.8%)	
**Length of stay (days)**	9 (6–13)	9 (6–13)	8 (4–13)	**< 0.001**
**CAR**	0.22 (0.07–0.48)	0.21 (0.06–0.45)	0.38 (0.15–0.72)	**< 0.001**
**GPS, n (%)**				**< 0.001**
** 0**	751 (23.4%)	704 (25.5%)	47 (10.4%)	
** 1**	1,423 (44.4%)	1,233 (44.7%)	190 (42.2%)	
** 2**	1,032 (32.2%)	819 (29.7%)	213 (47.3%)	
**mGPS, n (%)**				**< 0.001**
** 0**	2,052 (64%)	1,828 (66.3%)	224 (49.8%)	
** 1**	122 (3.8%)	109 (4%)	13 (2.9%)	
** 2**	1,032 (32.2%)	819 (29.7%)	213 (47.3%)	
**hs-mGPS, n (%)**				**< 0.001**
** 0**	494 (15.4%)	472 (17.1%)	22 (4.9%)	
** 1**	844 (26.3%)	759 (27.5%)	85 (18.9%)	
** 2**	1,868 (58.3%)	1,525 (55.3%)	343 (76.2%)	
**CRP (mg/L)**	6.6 (2.2–13.6)	6.2 (1.9–12.6)	10.1 (4.4–17.8)	**< 0.001**
**Albumin (g/L)**	31 (27–35)	31 (27–35)	27 (24–32)	**< 0.001**
**eGFR (BIS1, mL/min)**	42.27 (29.13–56.98)	43.55 (30.50–57.53)	34.84 (22.16–52.31)	**< 0.001**
**NLR**	7.47 (4.26–13.52)	6.90 (4.02–12.63)	11.03 (6.04–20.31)	**< 0.001**
**AST (U/L)**	19(14–31)	19(14–30)	22(14–38)	**0.006**
**ALT (U/L)**	14 (10–25)	14 (10–25)	16 (10–26)	0.102
**AST/ALT, median (IQR)**	1.23(1–1.6)	1.22(1–1.6)	1.28(1–1.7)	**0.014**
**RDW-SD (fL)**	48.8(45.5–53.3)	48.5(45.3–52.8)	50.8(47.1–56)	**< 0.001**
**RDW-CV (%)**	15.3(14.2–16.6)	15.1(14.1–16.4)	16.1(14.9–17.5)	**< 0.001**
**Ferritin (ng/mL)**	305(147–583)	293(139–549)	434(221–841)	**< 0.001**
**Hemoglobin (g/dL)**	11 (9.7–12.4)	11 (9.7–12.4)	10.65 (9.5–12.2)	**0.004**
**Platelets (n/mm**^**3**^**)**	210 (162–280)	211 (163.5–279.5)	208 (150–287)	0.359
**Use of 5 + Drugs, n (%)**	2,179 (68.0%)	1,914 (69.5%)	265 (58.9%)	**< 0.001**
**3 + lost ADL, n (%)**	2,206 (68.8%)	1,798 (65.2%)	408 (90.7%)	**< 0.001**
**3 + lost IADL, n (%)**	1,713 (53.4%)	1,440 (52.3%)	273 (60.7%)	**< 0.001**
**Comorbidities**				
** AMI, n (%)**	415 (12.9%)	352 (12.8%)	63 (14.0%)	0.472
** Liver cirrhosis, n (%)**	48 (1.5%)	44 (1.6%)	4 (0.9%)	0.252
** CHF, n (%)**	300 (9.4%)	232 (8.4%)	68 (15.1%)	**< 0.001**
** CeVD, n (%)**	498 (15.5%)	425 (15.4%)	73 (16.2%)	0.663
** Dementia, n (%)**	1,319 (41.1%)	1,125 (40.8%)	194 (43.1%)	0.360
** COPD, n (%)**	694 (21.6%)	623 (22.6%)	71 (15.8%)	**0.001**
** Parkinson, n (%)**	195 (6.1%)	171 (6.2%)	24 (5.3%)	0.473
** Hypertension, n (%)**	1,808 (56.4%)	1,602 (58.1%)	206 (45.8%)	**< 0.001**
** CKD, n (%)**	994 (31%)	884 (32.1%)	110 (24.4%)	**0.001**
** Diabetes, n (%)**	637 (19.9%)	570 (20.7%)	67 (14.9%)	**0.004**
** Sepsis, n (%)**	589 (18.4%)	434 (15.8%)	155 (34.4%)	**< 0.001**
** Cancer, n (%)**	298 (9.3%)	236 (8.6%)	62 (13.8%)	**< 0.001**

Data are median (IQR) for continuous variables or number (%) for categorical variables. *p*-values for Chi-squared tests. In bold significant associations

*P*-values for Chi-square (categorical variables) and Wilcoxon rank-sum (continuous variables) tests. Significant *p*-values are in bold

*ADL* Activities of daily living, *AMI* Acute myocardial infarction, *AST* Aspartate transaminase, *ALT* Alanine transaminase, *CAR* C-reactive protein to albumin ratio, *CeVD* cerebrovascular disease, *CHF* Congestive heart failure, *CFS* Clinical frailty scale, *CKD* Chronic kidney disease, *COPD* Chronic obstructive pulmonary disease, *CRP* C-reactive protein, *eGFR* Estimated glomerular filtration rate, *GPS* Glasgow prognostic score, *Hgb* Hemoglobin, *hs-mGPS* High sensitivity modified Glasgow prognostic score, *IADL* Instrumental activities of daily living, *mGPS* Modified Glasgow prognostic score, *NLR* Neutrophil-to-lymphocyte ratio, *RDW-SD* Red blood cell distribution width-standard deviation, *RDW-CV* Red blood cell distribution width-coefficient of variation

**Fig. 1:**
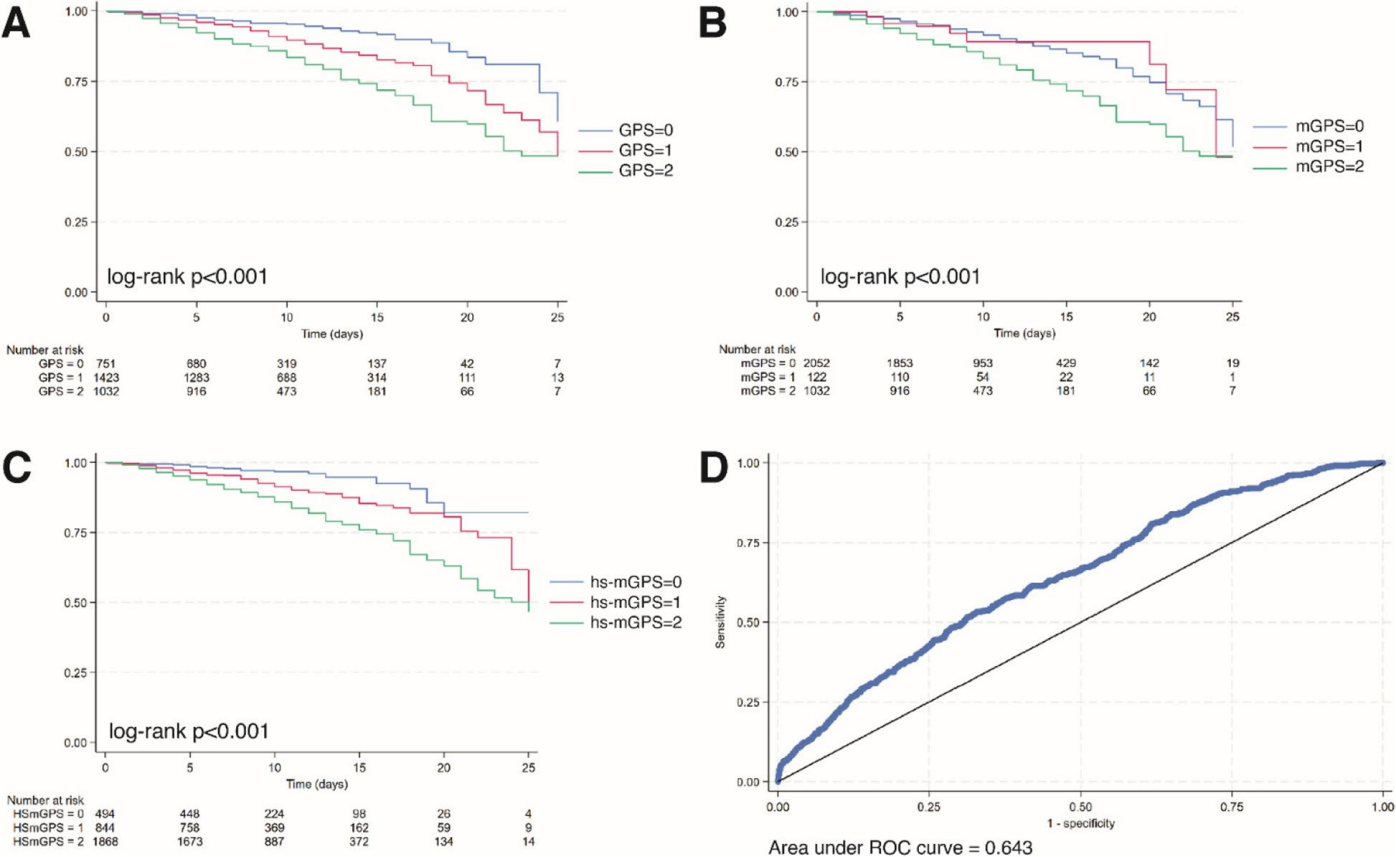
Kaplan-Meier survival estimates for (**A**) GPS, (**B**) mGPS, (**C**) hs-mGPS. **D** ROC curve for CRP-to-albumin ratio

The Kaplan–Meier analysis showed that patients in the highest scores of GPS, mGPS, and hs-mGPS have an increased mortality at 25 days from the hospitalization than patients with the lowest scores, with GPS and hs-mGPS achieving the best stratification (Fig. 1a, b, c). Regarding CAR, an AUC of 0.643 was obtained (Fig. 1d). The optimal cut-off for discriminating subjects according to their survival status was 0.35, with a sensitivity of 53% and a specificity of 63%, a positive predictive value of 21% and a negative predictive value of 90%.

In the survival analysis, the four CRP/albumin-related prognostic predictors were able to predict mortality in hospitalized patients. In a multivariable Cox proportional hazards model adjusted for age, gender, eGFR, NLR, ALT, Hgb, platelets, AMI, CHF, cerebrovascular disease, sepsis, dementia, COPD, Parkinson’s disease, hypertension, CKD, diabetes and cancer, CAR, GPS, mGPS, and hs-mGPS were significantly associated with mortality (Table 3). hs-mGPS showed the best discrimination ability, with scores greater than 0 associated with the highest effect sizes. Regarding the continuous index CAR, each unit increase was associated with a 2.9-fold increased risk of death (Table 3).

**Table 3:** Cox proportional hazards models (*N* = 3,206)

	**Model1**	**Model2**	**Model3**
	**HR (95%CI)**	**HR (95%CI)**	**HR (95%CI)**
**CAR**	**2.90 (2.40–3.49)**	**2.85 (2.36–3.45)**	**1.94 (1.56–2.41)**
**GPS**			
** 1**	**1.93 (1.40–2.65)**	**1.81 (1.32–2.50)**	1.36 (0.97–1.88)
** 2**	**3.24 (2.36–4.45)**	**2.99 (2.19–4.11)**	**1.91 (1.36–2.67)**
**mGPS**			
** 1**	0.97 (0.56–1.70)	1.02 (0.58–1.78)	1.15 (0.65–2.05)
** 2**	**1.99 (1.65–2.40)**	**1.91 (1.59–2.31**)	**1.50 (1.23–1.83)**
**hs-mGPS**			
** 1**	**2.24 (1.40–3.58)**	**2.19 (1.37–3.50)**	**1.79 (1.11–2.87)**
** 2**	**3.99 (2.59–6.14)**	**3.71 (2.40–5.71)**	**2.30 (1.47–3.59)**

In bold significant associations

Model 1: unadjusted

Model 2: adjusted for age, gender

Model 3: adjusted for age, gender, use of 5 + Drugs, 3 + lost ADL, 3 + lost IADL, eGFR, NLR, ALT, Hgb, Platelet, AMI, liver cirrhosis, CHF, CeVD, dementia, COPD, Parkinson, hypertension, CKD, diabetes, sepsis, cancer

In the fully adjusted survival model, we observed that the CRP/albumin-based biomarkers retain their prognostic value also in the presence of NLR as a confounder, which we previously identified as a strong predictor of in-hospital mortality in the same cohort [31]. CAR and NLR showed only a modest degree of reciprocal correlation (Spearman’s ρ = 0.26), allowing us to exclude multicollinearity issues in the model. In the fully adjusted model, increments of both indices were associated with increased mortality (Table S1). Moreover, the values for AIC and BIC were smaller for the fully adjusted models including both CAR and NLR (AIC, 6216.5 vs. 6193.5; BIC, 6368.3 vs. 6351.3), indicating a better fit. To further confirm the additive value of the two biomarkers, we compared the ROC curves and computed the overall net reclassification improvement (NRI). Significant ΔAUC of 0.030 (95% CI, 0.013 – 0.047) and overall NRI of 0.312 (95% CI, 0.217 – 0.408) were observed by adding NLR to CAR.

When considering males and females separately, we found that GPS and hs-mGPS provided a better stratification in males compared to females. Sex-specific Kaplan–Meier survival functions are reported in Fig. S1. Similarly, the Cox regression analysis evidenced a stronger association between CRP/albumin ratio-based indices (CAR, GPS, mGPS, and hs-mGPS) and mortality in males (Table S2), although significant associations persisted also in females. Notably, a 3.6- and 5.3-fold increased risk of death was observed in men with hs-mGPS of 1 and 2, respectively.

## Discussion

CRP is one of the most extensively studied nonspecific circulating biomarkers of inflammation. Although CRP was regarded as a passive, nonspecific marker of inflammation, increasing evidence indicates that CRP is a multifunctional player in the scenario of innate immunity [32]. Similarly, albumin can be considered an inflammatory marker because it acts as a negative acute-phase protein and its levels rapidly fluctuate during acute inflammation due to extracellular shifting [10]. The widespread use and applicability of more specific markers of inflammation is hampered by their availability in the clinical setting, therefore efforts have been devoted to identifying alternative biomarkers easily obtainable to be tested for predictivity. CRP-albumin ratio (CAR) is a novel prognostic biomarker predicted to be a more reliable indicator than CRP or albumin alone. In addition to CAR, other indexes based on CRP and albumin values have been described, such as GPS, mGPS, and hs-mGPS. All these biomarkers are produced by the integration of CRP and albumin laboratory indices, which independently serve as a convenient and cost-effective biomarker in routine clinical practice. Several studies have demonstrated that these parameters reflect the prognosis in patients with different types of ARDs, including cardiovascular diseases, diabetes, neurodegenerative diseases, and cancer (Carella et al., 2024 submitted). However, the prognostic relevance of these inflammatory-associated parameters in geriatric hospitalized patients has not been extensively investigated. Systemic inflammation is a feature of many acute conditions and is a strong predictor of adverse prognosis in adult and older patients [11]. We observed in a large real-world population of hospitalized older patients that CAR, GPS, mGPS, and hs-mGPS were significantly associated with a twofold increased risk of death, even after adjusting for various confounding variables. Interestingly, a hs-mGPS of 2 showed the highest effect size. In our cohort, patients who died were significantly older and had a higher prevalence of several chronic diseases, including CHF, COPD, CKD, Hypertension, Diabetes and Cancer, compared to those who survived. Therefore, our result confirmed and extended the results previously reported on the association of CRP-albumin-based parameters, especially CAR, with mortality in older patients selected for specific diseases, such as acute and chronic heart failure [33], acute kidney injury (AKI) [34], chronic obstructive pulmonary disease (COPD) [35], Parkinson’s disease [36] and different types of cancers [37, 38]. Notably, in our cohort, we demonstrated that the predictive value of CRP/albumin-based markers is independent of sepsis, which inherently induces strong elevations of CRP and a marked decline in albumin due to leakage into the extracellular fluid [39]. Although our results were based only on a single measure collected at the time of admission, longitudinal measures of CRP and albumin may provide useful estimates to track mortality risk in patients with multimorbidity over longer time spans. Indeed, a recent study investigating the sixth, third, and first month before mortality due to any cause in patients registered to the home health care services unit evidenced that significant increases in CAR between the premortem first- and sixth-month time points were significantly associated with mortality [40].

Interestingly, some studies suggested that the concurrent high levels of NLR and CAR values were more effective in predicting in-hospital mortality in older patients admitted to the emergency department (ED), compared to a separate evaluation [41]. In our cohort, we found that only a modest reciprocal correlation exists between CAR and NLR and that both indicators are independent predictors of mortality, with an additive value. In particular, NLR reflects bone marrow function and the balance between innate and adaptive immunity [42], while CAR provides a reliable quantitative measure of the intensity of the acute phase response [43]. Our results suggest that the two parameters describe aspects of systemic inflammation that partially overlap and encourage their incorporation in feasible scores for a comprehensive assessment of mortality risk in older patients.

Importantly, when the analysis was performed separately in men and women a stronger association with all the CRP-albumin-based parameters and mortality was observed in men, suggesting a gender-specific relevance of inflammation-based circulating parameters with mortality, in line with previous findings [23]. The mortality of older adults is sex-dependent, with the male gender showing a higher mortality risk than the female counterpart [44]. In our cohort of hospitalized patients, the in-hospital mortality is not different between men and women. However, the higher association of CRP-albumin-based parameters with mortality in men suggests a stronger contribution of systemic inflammation to adverse outcomes in men compared to women. Sex-based differences in susceptibility to a wide variety of diseases are increasingly recognized as an effect modifier in the interpretation of clinical trials and responses to medication, with consequences on therapeutic decisions. Indeed, it has long been observed that females are relatively protected from certain diseases characterized by systemic inflammation, which is mediated, at least in part, by oestrogen [45]. However, even if at the oldest age the effects of oestrogens are blunted, increased inflammatory responses could characterize old males compared to old females [46]. Notably, interleukin-6 (IL-6), a prototypical biomarker of systemic inflammation, has been reported to be chronically up-regulated in older males compared to females [47]. Furthermore, a comprehensive epigenetic study showed a higher innate immune and pro-inflammatory activity and lower adaptive immune activity in men aged > 65 years compared to age-matched women [48]. Taken together, these data support the notion that genetic and epigenetic mechanisms influence sex-related differences in immunosenescence and inflammaging. Further studies are needed to clarify the molecular basis of these differences.

Several limitations should also be addressed. First, our analysis was based on variables collected at the time of admission. Scores based on albumin and CRP may be affected by rapid variations of the two biomarkers that occurred during the hospitalization. Second, we limited the number of confounders that were included in the analysis to ensure the greatest sample size and avoid incomplete cases. Third, the lack of data on patients who did not provide informed consent prevented us from evaluating differences between participants and non-participants. As a result, we were unable to comment on the potential direction of selection bias, which limits the generalizability of our findings. However, these limitations are balanced by the prospective design of the study, the very high estimated participation rate (> 95%), and the large sample size, which included all patients admitted to the hospital during the observation period.

In conclusion, we showed that scores based on the cost-effective assessment of serum CRP and albumin offer additional guidance for stratifying in-hospital mortality risk in older patients, regardless of the diagnosis. The integration of laboratory biomarkers into multidimensional prognostic scores holds the potential to provide additional information reflecting the degree of innate immune activation and systemic inflammation in the clinical assessment of geriatric patients.

## Supplementary Information

Supplementary Material 1.

## Data Availability

No datasets were generated or analysed during the current study.
