# Correction: Inflammation scores based on C‑reactive protein and albumin predict mortality in hospitalized older patients independent of the admission diagnosis

**DOI:** 10.1186/s12979-024-00483-8

**Published:** 2024-11-13

**Authors:** Mirko Di Rosa, Jacopo Sabbatinelli, Angelica Giuliani, Miriam Carella, Daniele Magro, Leonardo Biscetti, Luca Soraci, Francesco Spannella, Massimiliano Fedecostante, Federica Lenci, Elena Tortato, Lorenzo Pimpini, Maurizio Burattini, Sara Cecchini, Antonio Cherubini, Anna Rita Bonfigli, Maria Capalbo, Antonio Domenico Procopio, Carmela Rita Balistreri, Fabiola Olivieri

**Affiliations:** 1 Centre for Biostatistics and Applied Geriatric Clinical Epidemiology, IRCCS INRCA, Ancona, Italy; 2 Department of Clinical and Molecular Sciences, Universita Politecnica Delle Marche, Ancona, Italy; 3 Clinic of Laboratory and Precision Medicine, IRCCS INRCA, Ancona, Italy; 4 Istituti Clinici Scientifici Maugeri IRCCS, Cardiac Rehabilitation Unit of Bari Institute, Bari, Italy; 5 Complex Operative Unit of Clinical Pathology, ARNAS Civico Di Cristina e Benfratelli Hospitals, Palermo, Italy; 6 Cellular, Molecular and Clinical Pathological Laboratory, Department of Biomedicine, Neuroscience and Advanced Diagnostics (Bi.N.D.), University of Palermo, Palermo, Italy; 7 Unit of Neurology, IRCCS INRCA, Ancona, Italy; 8 Unit of Geriatric Medicine, IRCCS INRCA, Cosenza, Italy; 9 Internal Medicine and Geriatrics, IRCCS INRCA, Ancona, Italy; 10 Geriatria, Accettazione Geriatrica e Centro Di Ricerca Per L’invecchiamento, IRCCS INRCA, Ancona, Italy; 11 Unit of Nephrology and Dialysis, IRCCS INRCA, Ancona, Italy; 12 Diabetology Unit, IRCCS INRCA, Ancona, Italy; 13 Cardiology Unit, IRCCS INRCA, Ancona, Italy; 14 Internal Medicine Department, IRCCS INRCA, Osimo, Italy; 15 Diagnostic Imaging, Clinical and Interventional Radiology, IRCCS INRCA, Osimo, Italy; 16 Scientific Direction, IRCCS INRCA, Ancona, Italy; 17 IRCCS INRCA, Ancona, Italy; 18 Advanced Technology Center for Aging Research, IRCCS INRCA, Ancona, Italy

**Correction: Immun Ageing 21**,** 67 (2024)**

10.1186/s12979-024-00471-y

Following publication of the original article [1], the authors reported that statement found in the Acknowledgements section is not correct.

The updated acknowledgement is given below.

**Acknowledgements**

This work was supported by Next Generation EU, in the context of the National Recovery and Resilience Plan, Investment PE8 – Project Age-It: “Ageing Well in an Ageing Society”. This resource was co-financed by the Next Generation EU [DM 1557 11.10.2022] to FO and ADP. The views and opinions expressed are only those of the authors and do not necessarily reflect those of the European Union or the European Commission. Neither the European Union nor the European Commission can be held responsible for them. This work was also supported by the Italian Ministry of Health (Ricerca Corrente to IRCCS INRCA).

The original article [1] has been updated.
