# Supplementary material for: Inflammation scores based on C-reactive protein and albumin predict mortality in hospitalized older patients independent of the admission diagnosis
Source: Immun Ageing. 2024 Oct 9;21:67. doi: 10.1186/s12979-024-00471-y (PMC11463076; doi:10.1186/s12979-024-00471-y)
Supplement: Supplementary file 1 — Supplementary Material 1. [file 12979_2024_471_MOESM1_ESM.pdf]

Inflammation scores based on C-reactive protein and albumin predict mortality in hospitalized older patients independent of the admission diagnosis

SUPPLEMENTARY MATERIAL

Figure S1. Kaplan-Meyer survival estimates for (A) Females and (B) Males.

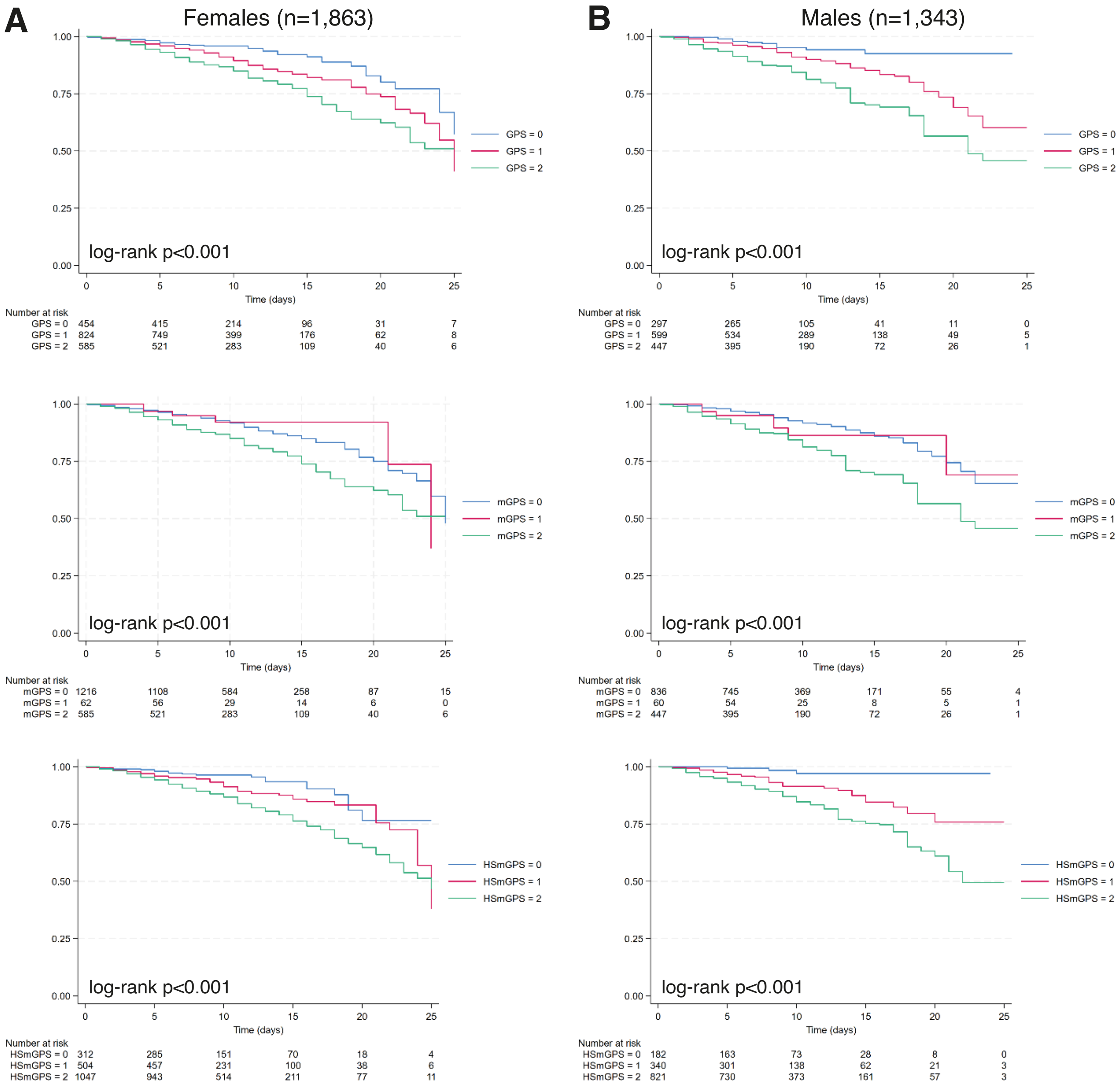

Table S1. Hazard ratios for all predictors included in the fully adjusted Cox proportional hazards model.

	**Model 3**
	**HR (95%CI)**
**CAR**	**1.94 (1.56-2.41)**
**Age**	**1.02 (1.00-1.04)**
**Female gender, ref. Male**	0.88 (0.72-1.06)
**5+ Drugs, ref. 0-4**	**0.69 (0.56-0.85)**
**3+ lost ADL, ref. 0-2**	**6.03 (3.79-9.58)**
**3+ lost IADL, ref. 0-2**	0.95 (0.73-1.23)
**eGFR (BIS1)**	**0.99 (0.98-0.99)**
**NLR**	**1.02 (1.01-1.02)**
**ALT**	**1.0007 (1.0003-1.0010)**
**Hgb**	1.02 (0.97-1.07)
**Platelets**	0.99 (0.99-1.00)
**AMI**	1.31 (0.99-1.74)
**Cirrhosis**	0.76 (0.28-2.04)
**CHF**	**1.90 (1.44-2.50)**
**Cerebrovascular diseas**	0.98 (0.76-1.27)
**Dementia**	**0.77 (0.63-0.95)**
**COPD**	0.90 (0.69-1.16)
**Parkinson**	1.06 (0.69-1.61)
**Hypertension**	0.85 (0.70-1.04)
**CKD**	**0.70 (0.56-0.89)**
**Diabetes**	0.99 (0.76-1.30)
**Sepsis**	**1.36 (1.10-1.69)**
**Cancer**	**1.67 (1.25-2.22)**

In bold significant associations.

Table S2. Sex-specific Cox proportional hazards models.

	**Females (n=1,863)**			**Males (n=1,343)**		
	**Model1**	**Model2**	**Model3**	**Model1**	**Model2**	**Model3**
	**HR (95%CI)**	**HR (95%CI)**	**HR (95%CI)**	**HR (95%CI)**	**HR (95%CI)**	**HR (95%CI)**
**CAR**	**2.45 (1.91-3.14)**	**2.39 (1.85-3.08)**	**1.48 (1.10-2.01)**	**3.81 (2.82-5.16)**	**3.73 (2.77-5.03)**	**2.68 (1.92-3.74)**
**GPS**						
**1**	**1.79 (1.22-2.63)**	**1.73 (1.18-2.54)**	1.38 (0.93-2.06)	**2.37 (1.31-4.27)**	**2.17 (1.20-3.92)**	1.43 (0.77-2.63)
**2**	**2.68 (1.83-3.93)**	**2.47 (1.68-3.64)**	**1.56 (1.02-2.39)**	**4.57 (2.56-8.15)**	**4.22 (2.36-7.54)**	**2.56 (1.39-4.73)**
**mGPS**						
**1**	0.80 (0.35-1.81)	0.89 (0.39-2.02)	1.18 (0.52-2.71)	1.23 (0.57-2.66)	1.23 (0.57-2.66)	1.30 (0.57-2.97)
**2**	**1.75 (1.37-2.24)**	**1.66 (1.30-2.13)**	1.21 (0.92-1.60)	**2.35 (1.75-3.14)**	**2.30 (1.72-3.08)**	**1.92 (1.41-2.61)**
**hs-mGPS**						
**1**	**1.79 (1.06-3.02)**	**1.76 (1.04-2.97)**	1.54 (0.91-2.63)	**5.26(1.61-17.19)**	**5.11(1.56-16.71)**	**3.62(1.09-12.00)**
**2**	**2.96 (1.85-4.75)**	**2.77 (1.72-4.44)**	**1.78 (1.09-2.93)**	**10.31(3.29-32.31)**	**9.55(3.04-29.98)**	**5.32(1.66-17.02)**

In bold significant associations.
Model 1: unadjusted
Model 2: adjusted for age
Model 3: adjusted for age, 5+ Drugs, 3+ lost ADL, 3+ lost IADL, eGFR, NLR, ALT, Hgb, Platelet, AMI, liver cirrhosis, CHF, CeVD, dementia, COPD, Parkinson, hypertension, CKD, diabetes, sepsis, cancer
